# The Ventilation of Hospitals

**Published:** 1887-04-23

**Authors:** C. Theodore Williams

**Affiliations:** Physician to the Hospital for Consumption and Diseases of the Chest, Brompton


### The Ventilation of Hospitals.

By C. Theodore Williams, M.A., M.D., F.R.C.P.,
Physician to the Hospital for Consumption and Diseases of the Chest, Brompton.

However important efficient ventilation is in our private dwellings, in our schools, our churches, our theatres, and our assembly rooms, where healthy people congregate, it is far more important in our hospitals, where, in addition to the ordinary risks incurred by placing a number of persons under the same roof, we have to deal with the products of various forms of disease, contagious and non-contagious, with accumulated secretions, with discharging wounds and ulcers, and with foul abcesses.

**Evils of Bad Ventilation.**

Although much may be done, and is done, to correct this by antiseptic measures we know too well that unless the air of a hospital be frequently renewed, we are pretty certain, in course of time, to make acquaintance with sore-throat, erysipelas, septicæmia, and hospital gangrene, and when these scourges have once obtained a footing, it is very difficult to dislodge them. Of the difficulty of getting rid of hospital gangrene, Hammond gives a good instance from the New York City Hospital records. Gangrene broke out in a ward, and the removal of the furniture and patients from the ward did not prevent a fresh set of patients being attacked. Closing the ward for some time, and whitewashing, had no effect; the plastering was then removed and fresh plaster applied, but still cases occurred. At last the entire ward was taken down and rebuilt, and then the plague was stayed. Similarly two hospitals in England—the Lincoln and the Norwich—in spite of all attempts at purification, became so stricken with pyæmia that they had to be pulled down and rebuilt.

**Hospitalism: Its Prevention.**

Sir James Simpson in his famous papers on Hospitalism, showed that the rate of mortality after amputations was greater in hospitals than in private practice, and this difference was more marked as the size of the hospital increased, the mortality rate being 1 in 9.2 for country operations, and 1 in 2.4 for hospital operations; and among the hospitals the rate varied according to the size, being 1 in 2.4 for hospitals containing 300 beds and upwards, and 1 in 6 for those with less than 100 beds. Sir J. Simpson dwelt much on the advantages of segregation of the sick over aggregation, and urged strongly the establishment of small cottage hospitals in place of the palatial buildings then existing, the dangers of which, however, he admitted might be met by really good ventilation. His statements were warmly contested by Mr. Holmes and others, and Mr. Erichsen, in his able work on Hospitalism, alleges as the cause of the high mortality in large hospitals the greater number of severe operations performed there, and also the bad construction of the old buildings, which, instead of being arranged on the pavilion, or separate-block system, were simply big houses, with basements containing kitchens, sculleries, cellars, and the ordinary offices of a large establishment, with an operating theatre and a mortuary, more or less connected with the main building, with every floor filled with sick and injured people; on the ground floor, accident and operation cases; on the first floor, probably medical cases; above, chronic surgical cases,—who can wonder, says Mr. Erichsen, at the development of pyæmia below, and of erysipelas above? The corridors and staircases were probably the channels of infection.

**Results of Antiseptic Surgery.**

Antiseptic surgery has worked wonders in many of our old hospitals, and enabled operations to be successfully performed, which otherwise could not have been attempted; but it must not be supposed that it does away with the necessity for good ventilation, and after all it is mostly applicable to surgical cases.

**Hospitals a Necessity.**

Hospitals of some size are a necessity, both to ensure good medical treatment and nursing, and to afford instruction and training to medical students; and though there may be some danger in the aggregation of the sick, this can for the most part be overcome by rigid attention to cleanliness, asepticism, and thorough ventilation. First, let us consider what kind of atmosphere we have to deal with in a hospital, and what impurities it is of importance to remove. The composition of pure air is, as we know:—Oxygen, 209.6 in 1,000 vols.; nitrogen, 790.; carbonic acid, .4; watery vapour, organic matter, ozone, salts of sodium, and other mineral salts, a variable amount. Ammonia a trace.

Now, in a hospital atmosphere, in addition to variations in the amounts of the above constituents, we are liable to impurities, through drainage, gas, and special organisms.

The numerous analyses made in the wards of hospitals show, that where the cubic space is insufficient the first element to increase is the carbonic acid (carbon dioxide). This in the best ventilated wards varies from .6 to .8 per 1,000; but in badly ventilated ones, rises to 1 and 2 per 1,000, according to Dr. de Chaumont's investigations. In some prisons he found it even exceeded 3.4; and between decks on board the *Alert*, during the Arctic expedition, Dr. Moss found it to amount to 4.8 when the hatches were down.

As the carbonic acid increases so the oxygen decreases but the organic matter appears to increase in the same proportion as the carbonic acid, and this gives a close atmosphere its peculiar odour. This odour generally appears when the amount of carbonic acid has reached .7 per 1,000; and from the organic matter which gives rise to it being slow to diffuse itself, is difficult to sweep away by ventilation, whereas the carbonic acid diffuses itself rapidly and thus is more easily got rid of. The organic matter is nitrogenous, and consists of various particles for most part suspended in the air. It is made up of cotton, woollen, and linen fibres, splinters from the floor; but also of hair, scales of epidermis, dried epithelial cells, pus cells, and often various organisms. Dr. de Chaumont found pus cells in the atmosphere of a ward of St. Mary's Hospital near some beds where erysipelas had prevailed. Dr. Bakewell detected the dried scales of small-pox in the air of a small-pox ward. Various bacteria have also been discovered, including the tubercle bacillus found by me in one of the extraction flues of the Derby Ward in this hospital. In hospitals for skin diseases the *achorion* has been noted near cases of favus, and the *trichophyton* in a ringworm ward.

Such being the case, we can quite understand how the contagion of certain diseases spreads; for, given an aggregation of the same cases, a number of solid particles, such as the epidermic scales from a scarlet fever or small-pox case, may be the vehicle of contagion in the same or in other wards. Dr. Percy Frankland, in some observations made in the Richmond Ward of the West wing of this hospital, found organisms present in the air at the rate of 43 volumes in ten litres of air, and a larger number when the patients were moving about and the windows were open; but the numbers were not so large as in the Natural History Museum on a visitors' day with the doors open.

**Ventilation of Drains.**

Another source of impurity in hospitals are the w.c.'s, sinks, and drains, which, unless well trapped and separately ventilated, may contribute noxious gases to the other contaminations of the air. In some of the continental hospitals it is hardly possible to stand in the middle of a large ward without being painfully aware of the locality and often of the proximity of closets and slop-sinks; and even in hospitals where powerful systems of ventilation are in use the very strength of the extracting current may be the means of drawing tainted air from these sources, unless a separate system of extraction has been arranged for them, which can always be done by placing them, as is the case here, in a block by themselves, provided with special extraction flues. Our first object in ventilation must be to sweep away the various impurities of the wards, or at least to dilute them to the utmost possible extent by introducing fresh air, while never neglecting scrupulous cleanliness both as regards patients and their surroundings, including careful removal of all dust and dirt. In this hospital, where a large proportion of the patients are consumptives, an element of danger is the expectoration, since this has been proved, even when dry and kept for months, to contain tubercle bacilli, still capable of cultivation in a suitable medium. Therefore the spittoons mixed with antiseptics are emptied twice a day into galvanised iron buckets, and stirred in with small coal into the furnaces used for heating the hospital, thus lessening the consumption of fuel, and also getting rid of a source of danger.

Cubic Space.

It is now generally admitted by the best authorities that while 500 cubic feet per head is sufficient for healthy people, 1,000 is none too much for sick ones, and that means should be taken to effect a change of atmosphere three times an hour. For nursing purposes a floor-space of at least ten square feet is advisable; wards should have a height of at least twelve feet, the other dimensions depending on the number of beds. In the new wing of this hospital the cubic space is 1,400 feet, the floor-space 116 feet, and the amount of fresh air supplied per hour upwards of 4,000 feet. This, too, does not include the large corridors and dining-halls, where the patients spend most of the day. In many hospitals the cubic space rises to 2,000 feet per patient, but it is seldom that so frequent a change of atmosphere is provided as in this hospital. The next question is the method of ventilation to be preferred—natural or artificial.

Natural Ventilation.

Natural ventilation, or the movements of the air due to diffusion of gases and differences in temperature between the inside and the outside of a room, may take place through a certain number of openings arranged as inlets and outlets in different parts of a room. Perforated bricks or wainscottings, holes in the ceiling, open windows, or glass louvres, and circular plates in windows, are examples; and so are Hinckes Bird's method of raising the window, and making the air pass between the two sashes, Tobin's tubes, Sherringham valves, and Boyle's inlets, all of which provide for the introduction of fresh air. Watson's and McKinnel's tubes are good instances of natural ventilation; but perhaps the most perfect is the force of the wind acting either by *persufflation*, *i.e.*, blowing through inlets, and even penetrating, as is shown by Pettenkofer, through brick walls, though checked by plaster; or acting by *aspiration*, *i.e.*, in blowing across an outlet, as a chimney, and thus giving rise to a current at right angles to itself up the chimney. Useful as *natural* ventilation is in the hospitals of warm climates, where patients can be fearlessly kept in thorough draughts, in temperate and cold climates we want to warm as well as to change the air, and we therefore have to adopt some form of *artificial* ventilation.

Extraction and Propulsion.

The two principal forms of artificial ventilation are ventilation by *extraction*, and ventilation by *propulsion*. In the former case either heat or an Archimedian screw may be used; in the latter, a propelling-fan or a bellows may be employed. In summer, when warming is unnecessary, one of the best forms of inlet for fresh air is the vertical or Tobin tube, rising to at least four, or possibly five feet in the ward, and placed either against the wall or in the centre of the ward, delivering air towards the ceiling and away from the patients. London smuts may be to a certain extent got rid of by this ingenious linen air-sieve arrangement of the Sanitary Engineering and Ventilation Company (115, Victoria Street). In winter, however, it is advisable either to heat this current, or to mingle warm air with it. In the French calorifers, used much on the Continent, the inlet tubes are heated by passing through ovens in the basement; but this system, though effective, causes a burnt smell in the wards from the combustion of any dust the air may contain. At the newly-erected Insel Spital, which is now outside the town of Berne, there is an ingenious arrangement of flues and valves by which the supply of hot and cold air can at any time be regulated to suit the outside temperature.

Another and better method is to bring the air over coils of hot-water-pipes, which do not cause burning of the dust, and enable us to introduce it from the same level as the ward, without passing through the basement. This is the plan pursued here. The air is admitted through gratings, and half passes between the hot-water-coils and half up a vertical tube, the streams mingling over the coil. The cold current, however, often maintains its vertical direction for a considerable distance, and leaves its mark on the ceiling above. The volume of air delivered by these tubes varies greatly according to the direction of the wind. If this blows towards the inlets the amount delivered is very large indeed. In the Maloja Hotel the incoming air is heated by passing between huge batteries of steam-pipes, and the extraction outlets are connected with the flues from the furnaces of the kitchens and bakeries.

Extraction by Heat.

The simplest form of extraction by heat is the ordinary fireplace and chimney, and a very effective extractor it has proved to be, especially if combined with a Boyle's valve, inserted near the ceiling. Some years ago, when, owing to leakage in the ventilation flues of the West wing of this hospital, the extraction rate was extremely feeble, amounting to scarcely one change of air in an hour, I found that with a good fire burning, the current up the chimney, as measured by the anemometer, was at the rate of 240 feet per minute, or sufficient to change the air $2\frac{1}{4}$ times an hour, thus supplying the deficiency in extraction. Even when there is no fire a fair current goes up the chimney.

Chimney Extraction.

With the chimney are connected various systems of extraction; some surround it with a second tube or chamber, into which the outlets from the rooms open, this being an economical method. Not uncommonly, as at St. Thomas's and Guy's, the extraction flues are brought into contact with the furnace chimneys, and thus utilise the heat of these latter. Extraction by heat is used most largely and effectively in mines and collieries, which are effectually ventilated by great furnaces kept burning at their mouths. The great fault of all extraction in connection with chimneys is that it varies with the amount of heat in them, and in the summer, when there is no fire, and owing to the possibility of open windows, the supply of fresh air is easy, the extraction of foul air, as important as ever, is at its lowest ebb.

System of the Brompton Hospital.

Extraction is performed in this hospital through towers containing coils of steam-pipes, of which there are four in this building. They are ranged one on either side of the entrance, and one in each wing. Our problem is to maintain a temperature of 62 deg. Fahr. day and night, and to change the air in each ward three times an hour. The wards are not large, and hold from one to eight patients. The inlets for warm and cold air above described, are placed immediately under the windows, and in the inside walls are the extraction flues running vertically with two openings, the upper one near the ceiling, the lower near the floor. They are 16 inches by 13 inches in size, and are covered by widely-meshed gratings. These flues join the main flues from the galleries, and open into the extraction shaft with as few curves and bends as possible. The principal extraction takes place through the top openings, which, according to my observations, draw two or three times as much as the lower ones, and it is of the utmost importance that the gratings, if any, should oppose as little obstruction to the current as possible. When testing the ventilation of the West wing I found that by removing the gratings, which are less perforated than those of the new building, I considerably increased the rate of the air current, showing that it was being retarded by friction, and consequently the gratings have been altogether removed from the top outlets of that wing. The temperature of the shaft is kept 10 deg. higher than that of the wards and galleries. In winter it is 72 deg. Fahr., but in summer it is kept much higher, as the temperature of the galleries rises, even though the warm coils are shut off. So much importance is attached to this point that it is my habit, and that of my colleagues, if during our visit we find two or three cases of sore-throat in a ward, to send for the engineer and ascertain if the heat of the shaft is properly maintained, as the purity of the air of the hospital depends on this. That extraction may go on, at any rate for a time, without this difference, however, is thus shown. A few years ago, during a severe winter, complaints were made that the ventilation of the old hospital did not proceed properly at night, from the furnace fires not being kept up, and the shaft falling in temperature. To test this, Dr. Reginald Thompson and I spent a night each taking observations on the two shafts and the galleries. We found that the temperature did fall at midnight, owing to want of attention to the stoking, and that for some time the heat did not exceed that of the wards, but that nevertheless the extraction current was maintained at a fair rate, in accordance, we supposed, with the first law of motion, that a body in motion will continue in uniform rectilinear motion, unless interfered with by some opposing force. However, to make matters safe, on our recommendation the committee of management provided that in future the heat should be as well kept up by night as by day.

Ventilation by *propulsion* consists of pumping air by means of a propelling fan or a bellows into a series of flues where it is heated and afterwards circulated. This system is in use at the Necker and Lariboisière Hospitals in Paris, where it acts successfully, and it is also to some extent used at St. George's Hall, Liverpool. The principle is that of the Indian punkah, and in this country the system is best adapted for quickly purifying large places of assembly, such as theatres and meeting halls. At one time the West wing of the Brompton Hospital was ventilated on this plan by a large pump, designed by Dr. Arnott, whose name is perpetuated in one of the wards. But, unfortunately, the machinery broke down, and in time the extraction system was substituted for it.

I will now sum up this paper with the following conclusions :—1. That to maintain the proper standard of purity in a hospital atmosphere in this climate, some form of artificial ventilation combined with warming the air is necessary. 2. That no system which does not provide for a change of air, at least three times an hour, outside the extraction by fireplaces, should be adopted. 3. That if extraction by heat be used, it is of the utmost importance that the temperature of the extracting shaft should be maintained as thoroughly in winter as in summer. 4. That the w.c.'s and slop-sinks of a hospital should be ventilated separately from the wards and corridors.

